# Chewing Lice (Phthiraptera: Amblycera, Ischnocera) of the Common Buzzards (*Buteo buteo*) in Romania: Host Age and Habitat Jointly Determine Lice Infestation

**DOI:** 10.3390/pathogens15020193

**Published:** 2026-02-10

**Authors:** Călin Mircea Gherman, Gianluca D’Amico, Katarzyna Anna Hołówka, Florinel Gheorghe Brudaşcă, Petru Burduhos, Alexandru Bulacu, Dan-Traian Ionescu, Sándor Hornok, Attila D. Sándor

**Affiliations:** 1 Department of Parasitology and Parasitic Diseases, University of Agricultural Sciences and Veterinary Medicine of Cluj-Napoca, Calea Mănăștur 3-5, 400372 Cluj-Napoca, Romania; calin.gherman@usamvcluj.ro (C.M.G.); katarzyna-anna.holowka@usamvcluj.ro (K.A.H.); 2 Department of Infectious Diseases, University of Agricultural Sciences and Veterinary Medicine of Cluj-Napoca, Calea Mănăștur 3-5, 400372 Cluj-Napoca, Romania; florinel.brudasca@usamvcluj.ro; 3 Department of Engineering and Environmental Protection, University of Agricultural Sciences and Veterinary Medicine of Cluj-Napoca, Calea Mănăștur 3-5, 400372 Cluj-Napoca, Romania; petru.burduhos@usamvcluj.ro; 4 Romanian Wilderness Society, Mihai Eminescu Street 98, 335500 Hateg, Romania; alexandru.bulacu@srswild.ro; 5 Department of Forestry, Faculty of Silviculture and Forest Engineering, Transylvania University, Șirul Beethoven Street 1, 500123 Brașov, Romania; dionescu@unitbv.ro; 6 Department of Parasitology and Zoology, University of Veterinary Medicine, István u. 2, 1078 Budapest, Hungary; hornok.sandor@univet.hu (S.H.); adsandor@gmail.com (A.D.S.); 7 HUN-REN Climate Change: New Blood-Sucking Parasites and Vector-Borne Pathogens Research Group, István u. 2, 1078 Budapest, Hungary; 8 STAR-UBB Institute, Babes-Bolyai University, Mihail Kogălniceanu Street 1, 400347 Cluj-Napoca, Romania

**Keywords:** ectoparasite, host–parasite relationships, mean intensity, prevalence, Phthiraptera

## Abstract

(1) Background: The common buzzard (*Buteo buteo*) is the most widespread raptor in Romania. This study aimed to assess the occurrence of chewing louse species and the factors influencing the epidemiology of louse infestation in the national bird populations. (2) Methods: Between 2012 and 2025, a total of 131 buzzards were collected from all over Romania, which were either roadkilled or died due to health issues. These birds were parasitologically examined, the gathered lice were identified, and epidemiological parameters were determined. (3) Results: The overall prevalence of louse infestation was 77.9%, with 4389 specimens collected. Five species were identified: *Degeeriella fulva* (55.7%), *Craspedorrhynchus platystomus* (37.4%), *Colpocephalum nanum* (42.0%), *Colpocephalum turbinatum* (7.6%), and *Laemobothrion maximum* (2.3%). Among the factors influencing the evolution of louse infestations, birds’ age statistically significantly affected only the mean intensity (48.0 in subadults and 28.6 in adults, *p* < 0.001). Combined origin and season through temperatures and relative humidity also influenced the mean intensity of infestations. Sex-ratio and nymph-to-female ratio were, in the majority, female-biased and nymph-biased. (4) Conclusions: Lice infestation patterns of common buzzards are shaped more commonly by environmental and biogeographic context than by host sex, with temperature, humidity gradients, and region of origin primarily influencing mean intensity rather than prevalence. In addition, sex ratios were consistently female-biased across all lice species, and nymph-to-female ratios suggested contrasting demographic trajectories among taxa, with evidence of expanding infrapopulations in some species and more senescent structures in others.

## 1. Introduction

The common buzzard (*Buteo buteo*) is one of the most widespread raptors in Europe, with the Romanian population being estimated between 20,000 and 50,000 breeding pairs [1]. The species is distributed across diverse range of habitats and exhibits considerable ecological plasticity. The species has probably the largest geographic range among *Buteo* hawks, covering the temperate belts of Europe and Western and Central Asia. It is the most common raptor of most deciduous or mixed forests, and is also regular in the intensively used mosaic habitats, too. The eastern populations (chiefly the *B. buteo vulpinus* subspecies) are largely distributed in the forests and true steppes of Europe and Asia. The latter are long-distance migrants (main wintering areas in the savanna region reaching even the Cape Province of South Africa), while the western and central European populations (mainly the *B. buteo buteo* form) are resident [2]. Romania lies at the limit of the distribution of the two forms, with breeding populations mainly recruited from the *B. buteo buteo* [3], while *B. buteo vulpinus* is commonly recorded in migration [4]. The species breeds primarily in twig nests built in higher trees and used for several years. They have a single brood of up to five siblings, with the young tended by both parents for several months [2]. As with the case of many other large-sized birds and especially the long-lived raptors, common buzzards may host several groups of internal and external parasites [3]. Within the varied parasitic fauna hosted by this species, chewing lice (Phthiraptera: Amblycera and Ischnocera) are particularly prevalent [5,6]. These insects are feather and skin debris feeders and may negatively impact their host’s condition by damaging feathers, thus impairing thermoregulation and flight capacity; subsequently, detrimental effects, such as increased predation risk, reduced body condition and lower survival rates, can occur [7]. Chewing lice are obligate parasites of warm-blooded vertebrates, with nearly 90% of all known species living on birds [8]. They show high host specificity, with extreme specialization to use a specific part of the hosts’ body. They are fully wingless, with propagation and new host colonization mainly during body contact between hosts belonging to the same species, in most bird species limited to the reproductive season [9]. Phoretic transfer using winged hippoboscid flies (Diptera: Hippoboscoidea) was recorded for certain lice species; however, it is rare [8].

Despite the ecological significance of host–parasite interactions, which include the potential to affect the health and fitness of their hosts by reducing the feather mass [10], causing intense pruritus, restlessness, irritation and trauma [6], the role as intermediate hosts for other parasitic infections [8], and affecting social interactions [11], relatively few studies have explored the diversity, intensity and prevalence of chewing lice in the common buzzard’s distribution area. Most studies relied on the identification and listing of the louse species found on common buzzards [12,13,14,15], with an extensive list collected along the species’ vast distribution range [9]. Research conducted over the last two decades has begun to address other questions, examining patterns of chewing louse infestation among wild raptors, considering host-related life history and environmental factors, such as host age, sex, body condition, environmental conditions [16], and migratory behavior [17]. However, most studies used either small numbers of birds [12] or relied heavily on birds in captivity [13,14,15], with all the known biases inherent in these studies [17]. Lice infestation of debilitated, recovering, injured raptors or birds in captivity may differ from that of naturally occurring individuals, due to parasite manipulation (medical treatment) or reduced capacity of antiparasitic defense by the host [17]. Several louse species have been recorded on common buzzards in Romania, with no information on host ecology or distribution [18,19,20,21,22,23]. Through parasitological screening of an extensive series of common buzzards of natural origin, this study aimed to identify several epidemiological parameters (prevalence, mean intensity, population sex ratio) of chewing lice parasitizing common buzzards in Romania. It also assessed the influence of various intrinsic (sex, age) and extrinsic (geographic origin, season) factors on the evolution of infestations. Based on collected data, we evaluated the impact that these key factors may play in driving louse prevalence in this common raptor.

## 2. Materials and Methods

### 2.1. Sample Collection and Origin

The birds were collected between 2012 and 2025 as part of an extensive study on the structure of the common buzzard’s helminth fauna [24,25,26,27]. They originated from all over Romania and were collected either as roadkill or after dying during emergency treatment of injured birds (most also victims of car traffic, some injured by hitting man-made objects in flight) at the Medical Clinic Department of Veterinary University.

Road-collected carcasses were sent to the Parasitic Diseases Department of the Faculty of Veterinary Medicine in Cluj-Napoca, and initially frozen at −18 °C. For each host, the exact collection date and location, mode of death, sex and age were established. Age was determined using the identification atlas of the continental birds of Southwestern Europe [28], and sex was identified during necropsy. Location was generated by extracting the digital georeferenced coordinates and altitude. The source for the altitude dataset was the CORINE Land Cover database, version 2016 (no climate-related data was used). This dataset is provided by the European Environment Agency (EEA, http://www.eea.europa.eu/, accessed on 1 October 2025).

### 2.2. Chewing Louse Collection and Identification

Following the birds’ thawing and before the parasitological necropsy, chewing lice were collected using the methods described by Jensen and Olsen [29], slightly modified by us.

First, the dry method was applied, consisting of vacuuming the birds’ feathers with a car vacuum cleaner. Each carcass was then held over a sheet of white paper, and the feathers were brushed by hand while the plumage was shuffled for a couple of minutes until no further debris or lice fell onto the paper. All dislodged material on the vacuum cleaner filter paper or white paper (e.g., feathers, sand, beach debris) was systematically searched for lice under a Olympus SZ51 (Olympus, Tokyo, Japan) dissecting microscope. Lice were collected with entomological forceps.

Secondly, the wet method (performed after the dry method) involved washing the same birds to recover any additional lice. Each carcass was thoroughly washed in a plastic bucket containing lukewarm water and liquid detergent and then left to soak in the same mixture for 24 h to facilitate lice detachment from the feathers. Afterwards, the water was strained through a 200 µm mesh sieve. Finally, each carcass was thoroughly rinsed in the bucket with a pressurized spray of water, and the rinse water was strained through the same sieve again. The retained material was then examined under a dissecting microscope, and any additional lice were collected.

All lice collected by these methods were stored in 70% alcohol and subsequently identified based on morphological descriptions published in various articles [9,30,31,32,33]. Each chewing louse was identified at the species level based on Price et al. [9], using an Olympus SZX16 Stereo Microscope (Olympus, Tokyo, Japan) and, for details, an Olympus BX-61 light microscope (Olympus, Tokyo, Japan), and images were taken using an Olympus SC180 camera (Olympus, Tokyo, Japan). Sex and developmental stage were individually recorded.

### 2.3. Statistical Procedures

Using the data collected from individual birds, we estimated the general and specific lice prevalence, infestation type (monospecific or polyspecific), population size, sex ratio expressed as the number of males per 100 females [(number of males/number of females) × 100] and as the ratio of males to females (number of males/number of females), and nymph-to-female ratio (nymph/female) of the pinpointed louse populations. Mean intensity, frequency, and prevalence, with their 95% confidence intervals (CIs), were calculated using the software Quantitative Parasitology 3.0 [34]. Prevalence-related data were compared using chi-square tests (or Fisher’s exact test, when sample sizes were small). The relationships between lice infestation intensity and biotic (age and sex of birds) and environmental (altitude) predictors were tested using Mann–Whitney U-tests. All statistical differences were considered significant for *p* < 0.05.

## 3. Results

**Figure 1:**
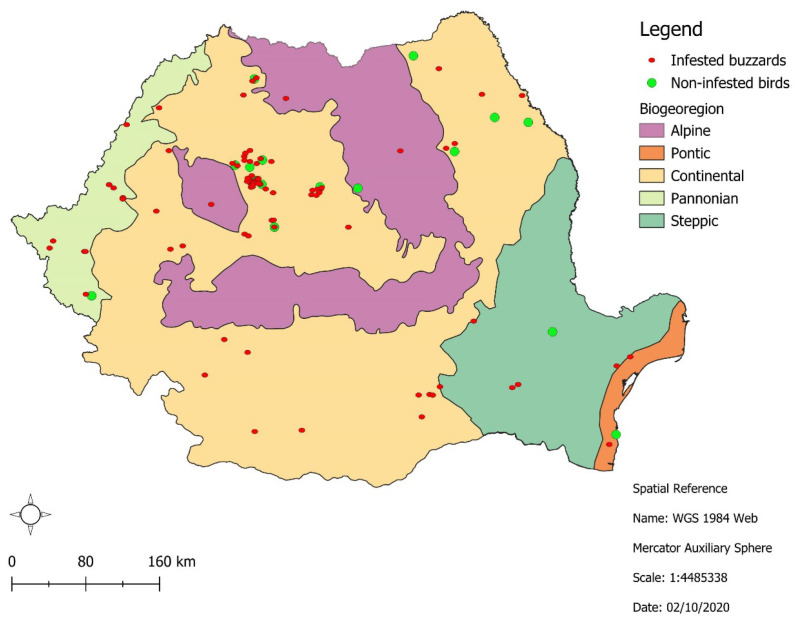
Origin of the common buzzards (*Buteo buteo*) sampled in Romania.

Between 2017 and 2025, a total of 131 common buzzard carcasses were collected across Romania (Figure 1). Of these, most were in the 2nd-year autumn/3rd-year spring age group (57/131; 43.5%), and males predominated (68/131; 51.9%). The majority of buzzards originated from a higher altitude Continental bioregion (100/131; 76.3%), and nearly half were collected during winter (62/131; 47.3%) (Table 1).

The overall prevalence (95% CI) of louse infestation on common buzzards was 77.9% (70.0–84.1%), with 4389 specimens collected from parasitized birds. Female hosts tended to have a marginally higher prevalence of infection (82.5% [71.4–90.0%]) than males (73.5% [62.0–82.6%]), but the difference was not statistically significant (further n.s.) (Table 2). Mean intensity was similar between female and male buzzards (42.3 versus 43.8, respectively; n.s.). Lice were more common on subadults than on adults (82.6% [73.6–89.0%] versus 66.7% [51.0–79.4%], respectively; n.s.), with a significantly higher number of lice present on subadults than adults (mean intensity: 48.0 versus 28.6, respectively; z = 2.0654, *p* < 0.001) (Table 2).

Birds originating from lower elevations (Pannonian, Pontic, and Steppe regions: 78.6% [69.8–85.5%]) showed similar prevalence to those originating from higher altitudes (Alpine and Continental regions: 75.0% [56.6–87.3%]) but hosted a significantly lower intensity of parasites (31.3 versus 46.1, respectively; z = 0.6573, *p* = 0.05). The detailed distribution of prevalence and intensity across bioregions is shown in Table 2. This difference remained even after controlling for age and was present in all but a single louse species (*Co. turbinatum*).

The seasonal distribution of chewing louse infestation on common buzzards showed the highest prevalence (86.5% [72.0–94.1]) in autumn, with a mean parasitism intensity of 49.1 specimens per bird, while spring was at the opposite pole (70.6% [46.9–86.7]), with a mean intensity of parasitism of 41.5 specimens per bird (Table 2). Although prevalence and median intensity varied seasonally, these variations were not statistically significant.

**Figure 2:**
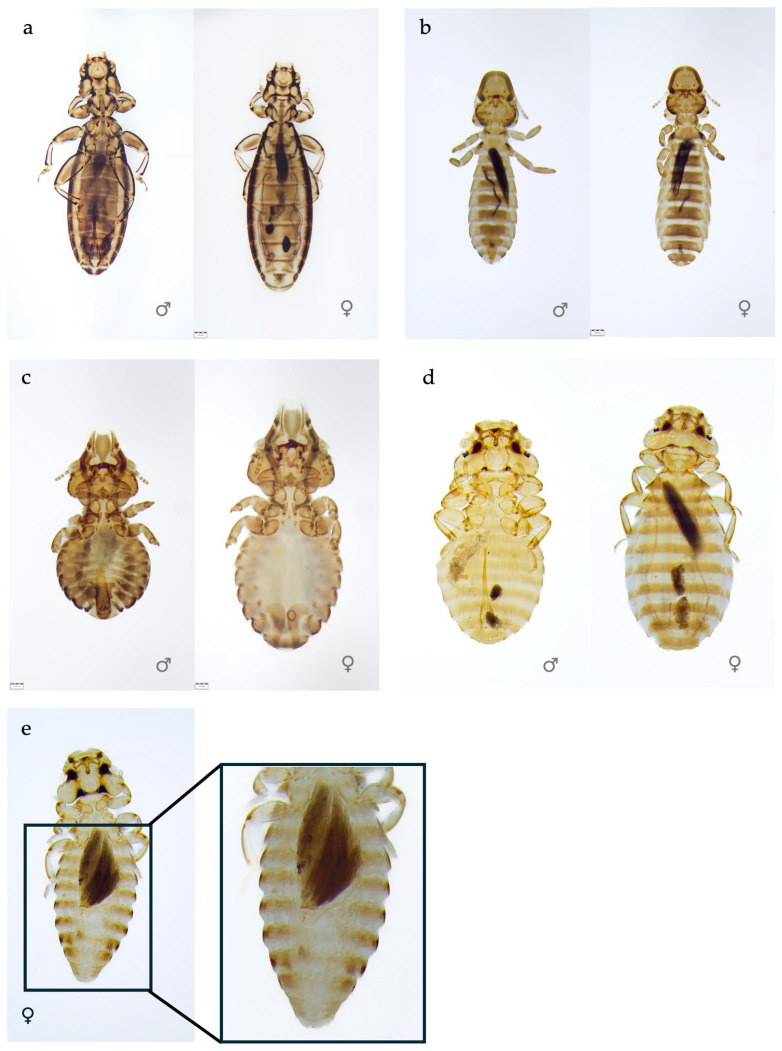
Chewing louse species identified in the common buzzard from Romania. (**a**). *Laemobothrion maximum*; (**b**). *Degeeriella fulva*; (**c**). *Craspedorrhynchus platystomus*; (**d**). *Colpocephalum nanum*; (**e**). *Colpocephalum turbinatum*.

A major difference was found in the prevalence of the five identified louse species, *Degeeriella fulva* (*D. fulva*) (parvorder Ischnocera), *Craspedorrhynchus platystomus* (*C. platystomus*) (parvorder Ischnocera), *Colpocephalum nanum* (*Co. nanum*) (parvorder Amblycera), *Colpocephalum turbinatum* (*Co. turbinatum*) (parvorder Amblycera), and *Laemobothrion maximum* (*L. maximum*) (parvorder Amblycera) (Figure 2).

The most common species was *D. fulva* with 2220 specimens collected (prevalence [95% CI]: 55.7% [47.2–63.9]); mean intensity: 30.4 specimens per bird, followed by *Co. nanum* with 599 specimens collected (prevalence: 42.0% [33.9–50.5]); mean intensity: 10.9 specimens per bird (Table 3).

*Craspedorrhynchus platystomus* affected 49 buzzards, from which 1176 lice were gathered (prevalence of 37.4%, [29.6–45.9]; mean intensity 24.0), while *Co. turbinatum* parasitized only 10 buzzards with 360 specimens collected (prevalence of 7.6%, [4.2–13.5], mean intensity 36.0). *Laemobothrion maximum* was found on only three birds, with 34 individuals gathered (prevalence of 2.3%, [0.8–6.5], mean intensity 11.3) (Table 3).

**Figure 3:**
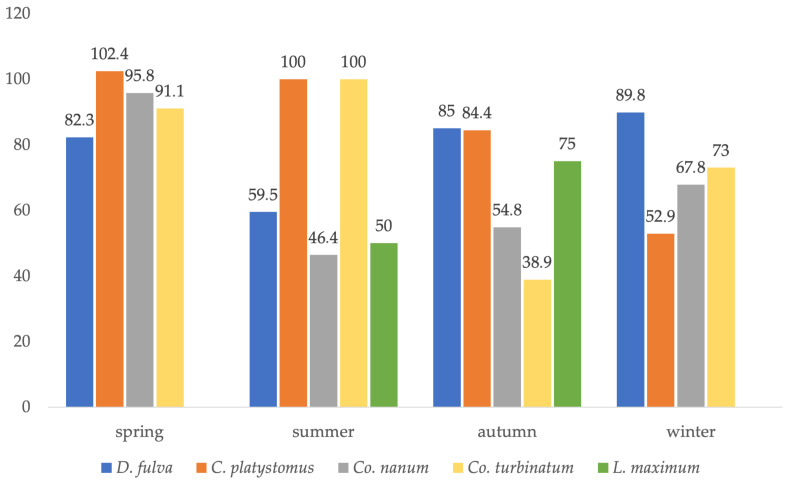
The seasonal variation in the sex ratio of chewing louse species parasitizing the common buzzard in Romania.

Overall, the sex ratio was female-biased (1:1.32 [75.4]), with Ischnoceran lice (*Degeeriella* and *Craspedorrhynchus* genera) recording a sex ratio of 1:1.30 (76.8), while Amblycerans (*Colpocephalum* and *Laemobothrion* genera) recorded 1:1.43 (69.8), a more pronounced female bias. Sex ratios varied among chewing louse species (64.2–82.3) but showed a consistent female bias across all species (Table 3). Seasonally, sex ratios by chewing louse species were below 100 in the majority of cases, indicating more females than males regardless of season, except for *C. platystomus*, which showed more males than females (102.4) in spring, and *Co. nanum* and *C. platystomus*, which showed an equal number of females and males in summer (100 each) (Figure 3).

The nymph-to-female ratio was nymph-biased for *C. platystomus* (1.20:1), *Co. turbinatum* (1.23:1), and *L. maximum* (4.0:1), and adult female-biased for *D. fulva* (0.51:1) and *Co. nanum* (0.88:1) (Table 3).

Mono- and polyspecific infections were observed, with associations of two, three, and even four louse species (Table 4). Co-occurrence of two or more louse species on the same host was relatively common, with 64.7% (95% CI: 55.1–73.3) of birds with lice harboring >1 louse species. More than two-thirds of buzzards (69.7% [57.8–79.4]) harbored two louse species, with the most common co-occurrence being *D. fulva*-*Co. nanum* (34.8% [24.5–46.9] of all co-parasitism cases), while more than a quarter (27.3% [18.0–39.0]) harbored three louse species. There were two outstanding cases (3.0% of all co-occurrences) with four louse species recorded on a single buzzard: an adult male with the simultaneous presence of *D. fulva*-*C. platystomus*-*Co. nanum*-*Co. turbinatum*, and a juvenile with *D. fulva*-*C. platystomus*-*Co. nanum*-*L. maximum* (Table 4).

Overall, Ischnoceran lice were more prevalent than Amblyceran lice (70.2% vs. 48.9%, *x^2^* = 12.4219, *p* < 0.001), with a higher mean intensity recorded on the birds, too (36.9 vs. 15.52, z = 4.083, *p* < 0.001). This was caused primarily by the higher Ischnoceran intensity of subadult birds (43.4 vs. 19.1, z = 2.9059, *p* = 0.0036), as there was no difference in the mean intensity among adults (14.7 vs. 17.8, n.s.). Dual infestation with lice from different parvorders (Amblycera and Ischnocera: 33 out of 66 cases of dual infestation) was significantly more common than with lice from the same parvorder (Amblycera-Amblycera: 1 or Ischnocera-Ischnocera: 12 out of 66 cases, Table 4) (Fisher’s exact test, *p* = 0.00045).

## 4. Discussion

### 4.1. Prevalence of Chewing Lice Infestation in the Species Distribution Areas

Large-scale, population-level studies of chewing louse in raptors are limited, with previous research mainly focusing on other raptor hosts from America [35,36] or Asia [17,37,38]. Outside the European distribution area, chewing lice on the common buzzard have been reported from Iran, where two additional species, *Degeeriella fusca* and *Cuclotogaster heterographus*, were identified compared with the present study [39]. In Africa, *L. maximum* has been reported from common buzzards in Egypt and appears to be the only species documented on the continent to date [40].

Within Europe, studies of common buzzards are also limited, likely due to the legal protection of raptors and logistical constraints on sampling [38]. The presence of *D. fulva*, *L. maximum*, *Co. nanum*, *Co. turbinatum*, *C. platystomus*, and *Kurodaia fulvofasciata* has been reported in common buzzards from Belgium [41]. In Spain, *L. maximum* recorded a prevalence of 21.8%, *D. fulva* 25.3%, *C. platystomus* 10.3%, and *Colpocephalum meridionale* (9.2%) [13]. In Portugal, the parasitism structure was similar to our study, but mean intensities of *D. fulva*, *G. platystomus*, and *L. maximum* infestations were lower than in the present study, with 1–2 specimens per bird [15]. In Italy, *D. fulva* (41.18%, 7/17), *Co. turbinatum* (11.76%, 2/17), and *C. platystomus* (5.88%, 1/17) have been reported in common buzzards [6], while in Hungary, *C. platystomus*, *Co. nanum*, *D. fulva*, *Menopon gallinae*, and *Lipeurus caponis* were identified [42]. In eastern Europe and adjacent regions, *Co. nanum*, *K. fulvofasciata*, *C. platystomus*, and *D. fulva* have been reported in common buzzards in Bulgaria [43], and *Laemobothrion circi*, *D. fusca*, and *C. platystomus* in Russia, in the North Caucasus [44]. In the Lower Russian Don region, the chewing lice parasitism structure was similar to ours: *Co. turbinatum*, *Co. nanum*, *C. platystomus*, *D. fulva*, and *L. maximum* [45]. In Turkey, *Co. nanum*, *Co. turbinatum*, *C. platystomus*, *D. fulva*, *Degeeriella nisus*, *K. fulvofasciata*, *L. maximum*, and *Falcolipeurus suturalis* have been recorded in buzzards from different regions [14,46,47,48,49,50,51].

Using mostly road-killed individuals, we sampled a large population of common buzzards in Romania to build a comprehensive dataset on host–parasite associations. Our findings are consistent with previous reports from Romania, where *Craspedorrhynchus dilatatus*, *C. platystomus*, *Co. nanum*, *Co. turbinatum*, and *D. fulva* have been identified in buzzards from different regions [18,19,20,21,22,23,52,53]. Notably, we report, for the first time in Romania, the presence of *L. maximum* on the common buzzard; this species is a fairly host-generalist louse that is frequently found on many large diurnal birds of prey [9].

### 4.2. Sex and Age Influence on the Prevalence of Infestations

Previous studies were generally descriptive, focusing on species identification, with limited information on prevalence and mean intensity, and very rare assessments of the influence of environmental or host-ecology-related factors on this ectoparasitism. In our study, the prevalence tended to be higher in females (82.5%) than in males (73.5%), and the intensity of parasitism was similar between sexes. Previous studies reported inconsistent influence of bird sex on chewing louse parasitism: some found no sex-associated differences [13], whereas others suggest that sex is less important than age and exposure. In the closely related accipitrid species, the rough-legged hawks (*Buteo lagopus*), females showed higher prevalence and intensity of louse infestations than males [54], aspect also recorded in the present study. Possible explanations include sexual size dimorphism, with females being larger than males across life stages [55], harboring larger infrapopulations of lice [56]. Additionally, limitations may occur in feather preening in females who become more active hunters as chicks grow, spending more time hunting and less time preening than males [57].

Similar to sex, age is a predictor that marginally influences the prevalence and mean intensity of chewing lice infestations in feathered raptors. This is confirmed in rough-legged hawks, where juvenile hawks had higher louse intensity and prevalence compared to adult hawks [54]. In Amur Falcons (*Falco amurensis*), the abundance of *Colpocephalum subzerafae* was influenced only by host age, being nearly four times higher in juveniles than in adults [38]. In contrast, common buzzards from Spain showed a higher prevalence of louse infestation in adults (42.2%) than in young birds (38.1%), but an opposite trend in mean intensity, with higher values in young birds (324) than in adults (254) [13]. Our study revealed a higher, although not statistically significant, prevalence in subadults (82.5%) and a significantly higher mean intensity (48.0) in subadults compared to adults (66.6% and 28.6). Subadult birds are more likely to be parasitized by chewing lice than adults because they are still developing their preening skills and are often in a more vulnerable state, such as recent fledging and less dense feathers [15]. Even if the chewing lice are not selective regarding younger or older feathers and primarily feed on the “fluffy” barbules and scales from any feather [58], higher prevalence and mean intensity in subadults can occur during the nestling period when lice are transferred from the female to the chicks. As the chicks grow, the female has time to preen her plumage, reducing the intensity of the infestation; meanwhile, the young have not yet developed these abilities, so both the prevalence and intensity will continue to increase in this age group. In addition to the mechanical defense against chewing lice, the birds’ immune system, particularly the T-cell-mediated response, can affect the diversity of lice they host and, in some cases, may trigger minor physiological reactions, such as an increase in eosinophils [59]. This is evident in amblyceran lice, which usually live in close contact with the host’s skin, feed on epidermal material and associated fluids, and may chew the tips of developing feathers to obtain access to blood or exudates. In contrast, ischnoceran lice live on plumage and feed on the non-living keratin of feather barbules. Møller and Rózsa [59] reported that amblyceran taxonomic richness is predicted by the intensity of T-cell-mediated immune responses in nestling hosts, whereas the T-cell response of adults had no significant effect. Conversely, ischnoceran taxonomic richness was not predicted by host T-cell responses, indicating that the birds’ immune system can mediate defense against chewing louse infestation. On the other hand, this feeding mode not only facilitates access of the host immune system to blood-feeding amblycerans but also implies that amblyceran and ischnoceran lice on the same host occupy different niches. This allows for their simultaneous occurrence more frequently than co-infections with lice that are taxonomically and ecologically more similar, as observed in our study [59].

### 4.3. Origin and Season Influence on the Prevalence of Infestations

Through climatic particularities, especially humidity and temperature, the birds’ origin and the collection season can influence the prevalence, but more importantly, the mean intensity of chewing louse infestations in feathered raptors. Among environmental conditions, relative humidity in the host’s habitat appears to strongly impact the mean intensity of lice, although its role remains debatable. Moyer et al. [60] found fewer lice on birds in arid regions, whereas Carrillo et al. [61] found high louse abundance in arid conditions. However, in nature, some species of lice are apparently excluded from hosts living in parts of their range where relative humidity is low [62,63]. Ischnoceran lice are less sensitive to low humidity than other taxa, as they can extract water from air at low relative humidity via extrusion of lingual sclerites on the hypopharynx [64]. This mechanism may help explain the large population sizes of *D. fulva* (2220 specimens) and *C. platystomus* (1176) recorded in our study compared to amblyceran populations observed on the same birds.

Temperature is another key factor shaping seasonal variation in the prevalence and intensity of chewing louse infections [65]. Similar to humidity, its effect is questionable, as lice can be abundant on birds living in environments with extremely low temperatures without limiting louse populations [66,67]. However, high temperatures combined with low humidity are considered detrimental to louse viability [68]. In our study, temperature appeared to influence infestation intensity, with the lowest mean parasitism intensity per bird recorded in summer (38.8) and the highest in autumn (49.1). Our results are in line with data from Spain, where seasonal prevalence in buzzards was the lowest (31.8%) in summer, while mean intensity was lowest in winter, corresponding to the hottest and coldest seasons, respectively [13].

When considering the combined effects of humidity and temperature on chewing lice, the birds’ origin significantly influenced mean intensity. In Romania, summer conditions in lower elevation regions are characterized by warm to hot average temperatures (20 to 26 °C) with relatively high humidity (RH 75–78%) [69,70], whereas winter average temperatures can drop below 0 °C (−15 to 1.5 °C) and RH increases to 82–88%. Under these conditions, prevalence did not vary substantially, but mean intensity was lowest in the two low-elevation, drier regions, ranging from 30.6 in the Steppe bioregion to 34.0 in the Pontic bioregion. By contrast, mean intensity was higher in the more humid, higher-elevation regions, reaching 45.5 in the Alpine bioregion and 46.1 in the Continental bioregion.

### 4.4. Sex-Ratio and Nymph-to-Female Ratio of Infestations

The sex ratio of raptor-infesting chewing lice reflects the relative numbers of males to females within a louse population on a given host and may be influenced by host-related factors such as sex, age, and body condition [71,72]. Although many ectoparasite groups tend to have even sex ratios [73], chewing lice can show skewed ratios, most commonly female-biased [74]. Our data support this aspect: all identified species showed female-biased sex ratios, with the lowest and highest values recorded among ischnocerans (1:1.21 in *D. fulva* and 1:1.55 in *C. platystomus*, respectively). However, few studies have examined sex ratios of chewing lice in feathered raptors, and we found no published data for the common buzzard, limiting direct comparisons. Nevertheless, in bald eagles, *Haliaeetus leucocephalus* (Accipitriformes: Accipitridae), examined in Manitoba, Canada, an opposite-biased sex ratio was established in two chewing louse species [75]. The ischnoceran *Degeeriella discocephalus* showed a slight male bias (sex ratio: 1.005, 2542 males versus 2529 females), while the amblyceran *Colpocephalum* sp. was slightly female-biased (0.997, 2942 males versus 2950 females). Still, skewed sex ratios, either male- or female-biased, have been reported frequently in chewing lice from non-raptor hosts [73,74].

The nymph-to-female ratio represents the proportion of nymphs relative to adult females within a population, reflecting the contribution of immature stages to the population structure and dynamics. Higher values indicate a younger lice population, whereas lower values suggest a senescence status of the respective population. The ratio is influenced by factors such as louse species, host characteristics, or mating competition [76]. In our study, the nymph bias observed among *C. platystomus*, *Co. turbinatum*, and *L. maximum* indicates growing infrapopulations, while the adult female bias observed for *D. fulva* and *Co. nanum* suggests aged, declining infrapopulations [71]. This parameter has been rarely investigated in feathered raptors. However, higher nymph-to-female ratios were recorded in bald eagles examined in Manitoba, Canada, for both *D. discocephalus* and *Colpocephalum* sp. [75]. Importantly, this study showed that the collection method influenced the estimated ratio: for *D. discocephalus*, the values were 3.42 with washing versus 2.12 with dry ruffling, and for *Colpocephalum* sp., the corresponding values were 3.26 and 2.28, respectively [75]. Seasonality may also affect nymph-to-female ratios, as demonstrated in owls in Canada, where the lice *Kurodaia magna* and *Strigiphilus remotus* recorded high nymph-to-female ratios during winter and warmer months [66].

## 5. Conclusions

Overall, this population-level study of common buzzards in Romania provides robust regional data on the structure of the chewing louse community in a widespread raptor and, for the first time, reports the presence of *Laemobothrion maximum* on this host in this country. Our findings suggest that lice infestation patterns of common buzzards are shaped more commonly by environmental and biogeographic context than by host sex, with temperature, humidity gradients, and region of origin primarily influencing mean intensity rather than prevalence. In addition, sex ratios were consistently female-biased across all lice species, and nymph-to-female ratios suggested contrasting demographic trajectories among taxa, with evidence of expanding infrapopulations in some species and more senescent structures in others. Together, these results highlight that chewing louse populations on common buzzards are conserved across regions, yet their population demography and infestation intensity can vary with ecological conditions, highlighting the value of standardized, population-level sampling for understanding ectoparasite dynamics in raptors.

## Figures and Tables

**Table 1:** Age, sex, origin, and seasonal distribution of the common buzzards collected from Romania (N-131).

Factor		Categories	n	%
Age	Subadults	Juvenile	35	26.7
		2YA/3YS	57	43.5
	Adults	3YA/4YS	21	16.0
		Adults	18	13.8
Sex	Males		68	51.9
	Females		63	48.1
Origin	Higher altitudes	Alpine	3	2.3
		Continental	100	76.3
	Lower elevations	Pannonian	18	13.8
		Pontic	2	1.5
		Steppe	8	6.1
Season	Spring (March, April, May)		17	13.0
	Summer (June, July, August)		15	11.5
	Autumn (September, October, November)		37	28.2
	Winter (December, January, February)		62	47.3

N—total number of common buzzards; n—number of common buzzards in a specific category; 2YA/3YS—2nd-year autumn/3rd-year spring; 3YA/4YS—3rd-year autumn/4th-year spring.

**Table 2:** Factors influencing chewing louse infestations in common buzzards from Romania.

Risk Factor			Frequency n/N	Prevalence % (95% CI)	(n’) Mean Parasitism Intensity/Bird
Sex	Males		50/68	73.5 (62.0–82.6)	(2190) 43.8
	Females		52/63	82.5 (71.4–90.0)	(2199) 42.3
Age groups	Subadults (Juvenile and 2YA/3YS)		76/92	82.6 (73.6–89.0)	(3645) 48.0
	Adults (3YA/4YS and Adults)		26/39	66.7 (51.0–79.4)	(744) 28.6
Origin	Alpine	81/103; 78.6% (69.8–85.5%)	2/3	66. 7 (20.8–93.9)	(91) 45.5
	Continental		79/100	79.0 (70.0–85.8)	(3641) 46.1
	Pannonian	21/28; 75.0% (56.6–87.3%)	13/18	72.2 (49.1–87.5)	(409) 31.5
	Pontic		1/2	50.0 (9.5–90.5)	(34) 34.0
	Steppe		7/8	87.5 (52.9–97.8)	(214) 30.6
Season	Spring		12/17	70.6 (46.9–86.7)	(498) 41.5
	Summer		12/15	80.0 (54.8–93.0)	(466) 38.8
	Autumn		32/37	86.5 (72.0–94.1)	(1571) 49.1
	Winter		46/62	74.2 (62.1–83.4)	(1854) 40.3

N—total number of common buzzards in a specific category; n—number of common buzzards with chewing louse infection in a specific category; n’—number of lice in a specific category; 2YA /3YS—2nd-year autumn/3rd-year spring; 3YA/4YS—3rd-year autumn/4th-year spring.

**Table 3:** Epidemiological indices of chewing louse species collected from common buzzards in Romania.

Species	Population						Frequency	Prevalence % (95% CI)	Mean Parasitic Intensity/Bird
	Size	M	F	N	SR	NtFR			
*D. fulva*	2220	781	948	491	82.3 (1:1.21)	0.51:1	73/131	55.7 (47.2–63.9)	30.4
*C. platystomus*	1176	265	413	498	64.2 (1:1.55)	1.20:1	49/131	37.4 (29.6–45.9)	24.0
*Co. nanum*	599	154	236	209	65.2 (1:1.53)	0.88:1	55/131	42.0 (33.9–50.5)	10.9
*Co. turbinatum*	360	94	119	147	79.0 (1:1.26)	1.23:1	10/131	7.6 (4.2–13.5)	36.0
*L. maximum*	34	4	6	24	66.6 (1:1.50)	4.0:1	3/131	2.3 (0.8–6.5)	11.3

M—males; F—females; N—nymphs; SR—sex ratio; NtFR—nymph-to-female ratio.

**Table 4:** Monospecific infestations and louse species associations in common buzzards from Romania.

Infestational Type		Frequency n/N	Prevalence % (95% CI)
Monospecific		36/102	35.3 (26.7–44.9)
Co-ocurrences	Associations	66/102	64.7 (55.1–73.3)
Two species	*D. fulva Co. nanum*	23/66	34.8 (24.5–46.9)
	*D. fulva C. platystomus*	12/66	18.2 (10.7–29.1)
	*C. platystomus Co. nanum*	5/66	7.6 (3.3–16.5)
	*D. fulva Co. turbinatum*	4/66	6.1 (2.4–14.6)
	*D. fulva L. maximum*	1/66	1.5 (0.3–8.1)
	*Co. nanum Co. turbinatum*	1/66	1.5 (0.3–8.1)
	Total	46/66	69.7 (57.8–79.4)
Three species	*D. fulva C. platystomus Co. nanum*	15/66	22.7 (14.3–34.2)
	*C. platystomus Co. nanum Co. turbinatum*	1/66	1.5 (0.3–8.1)
	*D. fulva C. platystomus L. maximum*	1/66	1.5 (0.3–8.1)
	*D. fulva C. platystomus Co. turbinatum*	1/66	1.5 (0.3–8.1)
	Total	18/66	27.3 (18.0–39.0)
Four species	*D. fulva C. platystomus Co. nanum L. maximum*	1/66	1.5 (0.3–8.1)
	*D. fulva C. platystomus Co. nanum Co. turbinatum*	1/66	1.5 (0.3–8.1)
	Total	2/66	3.0 (0.8–10.4)

N—total number of common buzzards in a specific category; n—number of common buzzards with monospecific or louse species associations in a specific category.

## Data Availability

The original contributions presented in this study are included in the article. Further inquiries can be directed to the corresponding author.

## References

[1] Societatea Ornitologică Română. https://pasaridinromania.sor.ro/specii/246/sorecar-comun-buteo-buteo.

[2] Cramp S., Simmons K.E.L. (1980). Handbook of the Birds of Europe the Middle East and North Africa: The Birds of the Western Palearctic: Volume II, Hawks to Bustards.

[3] Glutz von Blotzheim U.N., Bauer K., Bezzel E. (2001). Handbuch der Vögel Mitteleuropas, Band 4: Falconiformes–Greifvögel.

[4] Stanciu C.R., Zaharia R., Chișamera G.B., Cobzaru I., Gavril V.D., Murariu D. (2017). Migration Strategies of Common Buzzard (*Buteo buteo* Linnaeus, 1758) in Dobruja. Trav. Mus. Natl. Hist. Nat. “Grigore Antipa”.

[5] Stenkat J., Krautwald-Junghanns M.E., Schmidt V. (2013). Causes of morbidity and mortality in free-living birds in an urban environment in Germany. Ecohealth.

[6] Gherardi R., D’Agostino C., Perrucci S. (2021). Lice, Flies, Mites, and Ticks on Raptors (Accipitriformes, Falconiformes and Strigiformes) in Rescue Centers in Central Italy. Parasitologia.

[7] Barbosa A., Merino S., de Lope F., Møller A.P. (2002). Effects of Feather Lice on Flight Behavior of Male Barn Swallows (*Hirundo Rustica*). Auk.

[8] Clayton D.H., Adams R.J., Bush S.E., Atkinson C.T., Thomas N.J., Hunter D.B. (2008). Phthiraptera, the chewing lice. Parasitic Diseases of Wild Birds.

[9] Price R.D., Hellenthal R.A., Palma R.L., Johnson K.P., Clayton D.H. (2003). The Chewing Lice: World Checklist and Biological Overview.

[10] Booth D.T., Clayton D.H., Block B.A. (1993). Experimental demonstration of the energetic cost of parasitism in free-ranging hosts. Proc. Biol. Sci..

[11] Whiteman N.K., Parker P.G. (2004). Effects of host sociality on ectoparasite population biology. J. Parasitol..

[12] Demartis A.M., Restivo de Miranda M.A. (1978). Contributo alla studio dei Mallofagi di rapaci diurni. Gli. Uccelli D’Italia.

[13] Pérez J.M., Ruiz-Martínez L., Cooper J.E. (1996). Occurrence of chewing lice on Spanish raptors. Ardeola.

[14] Girisgin A.O., Dik B., Girisgin O. (2013). Chewing lice (Phthiraptera) species of wild birds in northwestern Turkey with a new host record. Int. J. Parasitol. Parasites Wildl..

[15] Tomás A., Palma R.L., Rebelo M.T., da Fonseca I.P. (2016). Chewing lice (Phthiraptera) from wild birds in southern Portugal. Parasitol. Int..

[16] Yamaç E., Dik B., Cavus M. (2023). The environment and host effects on chewing lice prevalence, richness, and abundance on birds in Turkey. Ornithol. Res..

[17] Yosef R., Strutzer O., Tabibi R., Rózsa L. (2019). Infestations of lice of steppe buzzards (*Buteo buteo vulpinus*) differ from those of common buzzards (*Buteo buteo buteo*). J. Raptor Res..

[18] Bechet I. (1961). Contributions to the Knowledge of Feather Lice from Romanian People’s Republic (sic). III.

[19] Rékási J., Kiss J.B. (1980). Weitere Beitrage zur Kenntnis der Federlinge (Mallophaga) der Vögel Nord-Dobrudscha (Rumanien). Parasitol. Hung.

[20] Adam C. (2003). Chewing lice (Phthiraptera: Amblycera, Ischnocera) collected from some bird species of Romania. Trav. Mus. Natl. Hist. Nat. “Grigore Antipa”.

[21] Adam C. (2007). Data on the chewing louse fauna (Phthiraptera: Amblycera, Ischnocera) from some Romanian autochthonous and exotic birds. Trav. Mus. Natl. Hist. Nat. “Grigore Antipa”.

[22] Adam C., Daróczi S. (2006). The chewing lice (Phthiraptera: Amblycera, Ischnocera) collected on some Falconiformes and Strigiformes (Aves) from Romania. Trav. Mus. Natl. Hist. Nat. “Grigore Antipa”.

[23] Rékási J., Kiss J.B., Sándor A.D. (2017). Chewing lice (Phthiraptera: Amblycera, Ischnocera) recorded from birds in the Danube Delta Biosphere Reserve: A literature review with new data. Aquila.

[24] Hołówka K.A., Ionică A.M., Ilea M., Poșa A.C., Cotuțiu V.D., Bulacu A., Sitko J., Vasiliu O.C., Mihalca A.D., Gherman C.M. (2024). Platyhelminthes of common buzzard (*Buteo buteo*): Checklist of species in Europe and new parasite-host associations in Romania. Int. J. Parasitol. Parasites Wildl..

[25] Hołówka K.A., Mihalca A.D., Ilea M., Poşa A.C., Vasiliu O.C., Bulacu A., Cobzaru I., Irimia A.G., Ionică A.M., Deak G. (2025). Nematodes and acanthocephalans of buzzards (*Buteo* spp.): Checklist of species in Europe and new host-parasite associations in Romania. Vet. Parasitol. Reg. Stud. Rep..

[26] Hołówka K.A., Negoescu A., Taulescu M., Ionică A.M., Deak G., Mihalca A.D., Gherman C.M. (2025). *Microtetrameres cloacitectus* in Eurasian buzzard (*Buteo buteo*): Pathology, phylogenetics, and seasonality. Parasitol. Res..

[27] Gherman C.M., Ionică A.M., Hołówka K.A., Cotuțiu V.D., Culda C.A., Lupu G.I., Mihalca A.D. (2025). *Oxyspirura petrowi* Causing Ocular Parasitism in a Free-Ranging Common Buzzard (*Buteo buteo*) in Romania and a Review of the Potential Zoonotic Implications as Cutaneous Larval Migrans. Animals.

[28] Blasco-Zumeta J., Heinze G.-M. (2023). Identification Atlas of the Continental Birds of Southwestern Europe.

[29] Jensen J.-K., Olsen B. (2003). A comparison of two methods for collecting feather lice from dead birds. Atlant Seab..

[30] Dik B., Halajian A., Turner M. (2013). The morphology of *Craspedorrhynchus platystomus* (Burmeister, 1838), a louse commonly found on the long-legged buzzard *Buteo rufinus* (Phthiraptera: Ischnocera: Philopteridae). Turk. J. Zool..

[31] Dik B., Halajian A., Turner M. (2018). Light Microscopy and Scanning Electron Microscopy of *Colpocephalum nanum* Piaget, 1890 (Phthiraptera: Amblycera: Colpocephalidae). Turk. Parazitol. Derg..

[32] Nelson R.C., Price R.D. (1965). The *Laemobothrion* (Mallophaga: Laemobothriidae) of the Falconiformes. J. Med. Entomol..

[33] Perez J.M., Granados J.E., Ruiz I. (1995). The morphology of *Laemobothrion* (*Laemobothrion*) *maximum* (Phthiraptera: Laemobothriidae). Parassitologia.

[34] Rózsa L., Reiczigel J., Majoros G. (2000). Quantifying parasites in samples of hosts. J. Parasitol..

[35] Órdenes M.J.L., Ibáñez C.B., Contreras R.L., Schmäschke R., Daugschies A., González-Acuña D. (2005). Ectoparasitismo en tiuque común *Milvago chimango chimango* (Vieillot, 1816) (Aves, Falconidae) en la zona de Ñuble, Chile. Lundiana.

[36] Liébana M.S., Santillán M.Á., Cicchino A.C., Sarasola J.H., Martínez P., Cabezas S., Bó M.S. (2011). Ectoparasites In Free-Ranging American Kestrels In Argentina: Implications for the Transmission of Viral Diseases. J. Raptor Res..

[37] Saxena A.K. (2017). Population characteristics of Black Kite lice. J. Parasit. Dis..

[38] Piross I.S., Siliwal M., Kumar R.S., Palatitz P., Solt S., Borbáth P., Vili N., Magonyi N., Vas Z., Rózsa L. (2020). Sex interacts with age-dependent change in the abundance of lice-infesting Amur Falcons (*Falco amurensis*). Parasitol. Res..

[39] Bahiraei Z., Sazmand A., Khedri J., Babaei M., Moeinifard E., Dik B. (2024). Chewing lice of wild birds in Iran: New data and a checklist of avian louse species reported in Iran. Front. Vet. Sci..

[40] Adly E., Gustafsson D.R., Nasser M.G., Baeshen R.S., Kamal M. (2022). Host–Parasite Associations and New Records of Chewing Lice (Phthiraptera: Amblycera, Ischnocera) from Raptors (Accipitriformes, Falconiformes, Strigiformes) Encountered in Egypt. J. Entomol. Sci..

[41] Hellenthal R.A., Price R.D., Palma R.L. Chewing Lice of Belgium. https://www.yumpu.com/it/document/view/11912389/chewing-lice-of-belgium-ronald-a-hellenthal1-roger-d-price2-.

[42] Solt S. (1998). Lice (Phthiraptera: Amblycera, Ischnocera) of raptors in Hungarian zoos and rehabilitation centers. J. Raptor Res..

[43] Ilieva M. (2009). Checklist of the chewing lice (Insecta: Phthiraptera) from wild birds in Bulgaria. Zootaxa.

[44] Lyakhova O.M., Kotti B.C. (2011). Chewing lice (Mallophaga: Insecta) of birds in the Central Ciscaucasia. Entmol. Rev..

[45] Malysheva O.D., Zabashta A.V., Tolstenkov O.O. (2018). To the fauna of chewing lice (Insecta: Phthiraptera) of birds (Aves: Falconiformes, Strigiformes) in the Lower Don region, Russia. Kavk. Èntomol. Bûll..

[46] Dik B., Yamac E. (2024). New data on the chewing lice (Psocodea: Phthiraptera) of domestic and wild birds in Türkiye. Vet. Parasitol. Reg. Stud. Rep..

[47] Dik B., Yamaç E., Uslu U. (2013). Studies on Chewing Lice (Phthiraptera: Amblycera, Ischnocera) Species from Domestic and Wild Birds in Turkey. Kafkas Univ. Vet. Fak. Derg..

[48] Dik B., Per E., Erciyas Yavuz K., Yamaç E. (2015). Chewing lice (Phthiraptera: Amblycera, Ischnocera) species found on birds in Turkey, with new records and a new host association. Turk. J. Zool..

[49] Dik B., Naz S., Sajid M.S. (2022). Data on the chewing lice (Phthiraptera) parasitizing the accipitrid birds (accipitriformes) in Turkey. J. Anim. Health Prod..

[50] Eren G., Özkoç Ö.Ü., Açici M. (2022). Contributions to the knowledge of the diversity of the chewing lice fauna in Turkey. Turk. J. Zool..

[51] İnci A., Dik B., Kibar M., Yildirim A., Düzlü Ö. (2010). Chewing Lice (Phthiraptera) Species on Wild Birds in Cappadocia Region, Turkey. Turk. Parazitol. Derg..

[52] Rékási J., Kiss J.B. (2006). New data on the lice (Phthiraptera) of some birds in Northern Dobrogea (Romania). Acrocephalus.

[53] Adam C., Chișamera G., Daróczi S.J., Sándor A.D., Gogu-Bogdan M. (2009). Data on the Chewing Louse Fauna (Phthiraptera: Amblycera, Ischnocera) from Some Wild and Domestic Birds of Romania. Trav. Mus. Natl. Hist. Nat. “Grigore Antipa”.

[54] Maron M.W., Paprocki N., Owen J.P., Conway C.J. (2024). Differential Effects of Chewing Lice on Body Condition across Host Age and Sex in Rough-legged Hawks (*Buteo lagopus*). J. Wildl. Dis..

[55] Ferguson-Lees J., Christie D.A. (2001). Raptors of the World.

[56] Durkin E.S., Luong L.T., Bird J. (2015). Mechanisms underlying parasite infection: Influence of host body mass and age on chewing louse distribution among brown-headed cowbirds. Parasitol. Res..

[57] Bush S.E., Clayton D.H. (2023). Does Preening Behavior Reduce the Prevalence of Avian Feather Lice (Phthiraptera: Ischnocera)?. J. Parasitol..

[58] Kaufman P.E., Koehler P.G., Butler J.F. (2007). External Parasites of Poultry: ENY-290 IG140.

[59] Møller A.P., Rózsa L. (2005). Parasite biodiversity and host defenses: Chewing lice and immune response of their avian hosts. Oecologia.

[60] Moyer B.R., Drown D.M., Clayton D.H. (2002). Low humidity reduces ectoparasite pressure: Implications for host life history evolution. Oikos.

[61] Carrillo C.M., Valera F., Barbosa A., Moreno E. (2007). Thriving in an arid environment: High prevalence of avian lice in low humidity conditions. Ecoscience.

[62] Bush S.E., Harbison C.W., Slager D.L., Peterson A.T., Price R.D., Clayton D.H. (2009). Geographic variation in the community structure of lice on western scrub-jays. J. Parasitol..

[63] Bush S.E., Waller M.M., Davis K.M., Clayton S.F., Clayton D.H. (2024). Birds in arid regions have depauperate louse communities: Climate change implications?. Ecol. Evol..

[64] Rudolph D. (1983). The water-uptake system of the Phthiraptera. J. Insect Physiol..

[65] Johnson K.P., Clayton D.H., Price R.D., Hellenthal R.A., Palma R.L., Johnson K.P., Clayton D.H. (2003). The biology, ecology, and evolution of chewing lice. The Chewing Lice: World Checklist and Biological Overview.

[66] Galloway T.D., Lamb R.J. (2019). Infestation parameters for chewing lice (Phthiraptera: Amblycera and Ischnocera) infesting owls (Aves: Strigidae) in Manitoba, Canada. Can. Entomol..

[67] Lamb R.J., Galloway T.D. (2016). Seasonal population dynamics of chewing lice (Phthiraptera: Amblycera and Ischnocera) infesting three species of woodpeckers (Aves: Piciformes: Picidae) in Manitoba, Canada. Can. Entomol..

[68] Derylo A. (1975). Investigation on economic disadvantage of Mallophaga IV. The influence of ecological and physiological factors on the intensity of Mallophaga infestation. Prz. Zool..

[69] Nicula G., Manafu A., Stanciu E. (2012). Natura 2000 în România.

[70] WorldData.info. https://www.worlddata.info/europe/romania/climate.php.

[71] Galloway T.D., Lamb R.J. (2021). Population Dynamics of Chewing Lice (Phthiraptera) Infesting Birds (Aves). Annu. Rev. Entomol..

[72] Pap P.L., Adam C., Vágási C.I., Benkő Z., Vincze O. (2013). Sex ratio and sexual dimorphism of three lice species with contrasting prevalence parasitizing the house sparrow. J. Parasitol..

[73] Marshall A.G. (1981). The sex ratio in ectoparasitic insects. Ecol. Entomol..

[74] Clayton D.H., Gregory R.D., Price R.D. (1992). Comparative ecology of Neotropical Bird Lice. J. Anim. Ecol..

[75] Lavallée C.D., Galloway T.D., Rochon K. (2020). Infestation parameters of chewing lice (Phthiraptera: Amblycera and Ischnocera) on bald eagles, *Haliaeetus leucocephalus* (Accipitriformes: Accipitridae), in Manitoba, Canada. Can. Entomol..

[76] Galloway T.D., Lamb R.J. (2015). Abundance and stability of populations of a chewing louse, *Mulcticola macrocephalus* (Phthiraptera: Philopteridae), on common nighthawks, *Chordeiles minor* (Caprimulgiformes: Caprimulgidae) in Manitoba, Canada. Can. Entomol..

